# Elevated circulating levels of gasdermin D are related to acute myocardial infarction and pyrogptosis

**DOI:** 10.1186/s12872-022-02998-8

**Published:** 2022-12-21

**Authors:** Yawen Weng, Bozhi Ye, Jiahui Lin, Shuang Lin, Lingfeng Zhong, Weijian Huang, Xueli Cai, Weiqi Wang

**Affiliations:** 1 Department of Cardiology, The Key Lab of Cardiovascular Disease of Wenzhou, The First Affiliated Hospital of Wenzhou Medical University, 2 Fuxue Road, Wenzhou, 325000 Zhejiang People’s Republic of China; 2 State Key Laboratory of Oncogenes and Related Genes, Stem Cell Research Center, Ren Ji Hospital, School of Medicine, Shanghai Cancer Institute, Shanghai Jiao Tong University, Shanghai, 200127 People’s Republic of China

**Keywords:** Gasdermin D, Acute myocardial infarction, Pyroptosis

## Abstract

**Background:**

Acute myocardial infarction (AMI) is one of the leading contributors to morbidity and mortality worldwide, with a prevalence of nearly three million people, and more than one million deaths reported in the United States every year. Gasdermin D (GSDMD) is involved in the development of atherosclerosis as a key protein of proptosis. This study was designed to determine the potential relationship of GSDMD with AMI in Chinese patients.

**Methods:**

One hundred patients with AMI and 50 controls were consecutively enrolled in this prospective observational study. GSDMD expression levels and other clinical variables in peripheral blood mononuclear cells (PBMCs) were measured upon admission to the hospital. All patients were followed up for 360 days, and the endpoint was considered the occurrence of major adverse cardiovascular events (MACE).

**Results:**

GSDMD expression levels in the PBMCs of patients with AMI were significantly higher than those in the controls. Moreover, our analysis showed that GSDMD was an independent biomarker of AMI and had a promising diagnostic ability for it. Finally, the results suggested that high expression of GSDMD and diabetes increased the risk of MACE after AMI.

**Conclusions:**

This study indicated that the GSDMD expression level in PBMCs was elevated in AMI patients and was closely associated with the pyroptosis of AMI.

## Introduction

Acute myocardial infarction (AMI) is one of the leading contributors to morbidity and mortality worldwide, with a prevalence of nearly three million people, and more than one million deaths reported in the United States every year [1, 2]. It is estimated that approximately 70% of fatal events in patients with AMI are related to the occlusion of atherosclerotic plaque. The early identification and clinical diagnosis of AMI are particularly critical because of the rapid myocardial ischemia caused by coronary plate occlusion. Over the past decade, cardiac troponin I(cTnI) remains the gold standard for the diagnosis of AMI, but elevated cTn is frequently observed in patients without clinical symptoms of AMI, often reflecting myocardial injury of “unknown origin” [3, 4]. Other clinical markers like CK-MB, heart-type fatty acid-binding protein(H-FABP) and myoglobin, still have defects such as low sensitivity, high time-specificity requirements, and limited detection methods [5, 6].

Gasdermin D (GSDMD), an emerging protein, has been regarded as one of the important mediators of pyroptosis [7, 8]. And pyroptosis represents a form of cell death that is triggered by proinflammatory signals and associated with inflammation, which has been reported to be involved in atherosclerosis by promoting the release of inflammatory factors [9]. The formation and rupture of arterial plaque are the main causes of AMI. At present, a large number of studies have shown that GSDMD, as the core protein of pyroptosis, plays an important role in the process of atherosclerosis. For example, a recent article published in nature showed in detail that downregulation of GSDMD expression in macrophages reduces macrophage proliferation and necrotic formation by inhibiting the inflammasome product interleukin-1β, thereby stabilizing plaques [10]. In addition, with the application of GSDMD^−/−^ mice, studies clarified that the reduction of GSDMD can effectively inhibit the level of inflammation and pyroptosis in mice, thereby improving the formation of arterial plaques and delaying atherosclerosis [11, 12]. Similarly, Mengqing et al. demonstrated that Caspase-11-gasdermin D-mediated pyroptosis is involved in the pathogenesis of atherosclerosis in vivo models [13]. Although a large amount of basic data reveal the role of GSDMD in atherosclerosis progression, there is still no clinical evidence to elucidate the association between GSDMD and AMI. Here, our study aimed to determine the relationship between the expression level of GSDMD and Chinese patients with AMI.

## Materials and methods

### Study population

This study was a prospective, single-center project. A total of 150 patients were enrolled from the Cardiology Department of the First Affiliated Hospital of Wenzhou Medical University, China, from October 2017 to August 2019. Patients were divided into three groups: the control group (n = 50), the group with AMI with single vessel disease (n = 40), and the group with AMI with multivessel disease (n = 60). By standard clinical practice, all patients underwent clinical evaluation, including laboratory examination (serum creatinine, C-reactive protein, NT pro-BNlow-density lipoprotein-cholesterol, etc.), echocardiogram examination, brain CT scan, 24-h Holter monitoring and/or 12-lead electrocardiograms (ECGs). In our study, AMI was diagnosed according to the presence of two of the following criteria: (a) prolonged chest pain; (b) increased troponin I level (> 0.15 µg/l); and (c) typical ECG changes, which included ST-segment elevation myocardial infarction and non-ST-segment elevation myocardial infarction. DAPT and statin were used in a standard manner for patients in all groups. Exclusion criteria included patients with acute or chronic inflammation, ischaemic stroke history, severe liver or renal dysfunction, hematological diseases, peripheral vascular disease, autoimmune disease, and cancer.

This study was approved by the Medical Ethics Committee of the First Affiliated Hospital of Wenzhou Medical College (China) and was performed according to the Declaration of Helsinki. Written informed consent was provided by every patient before enrolment in this study.

All patients underwent clinical follow-up for 365 days after discharge to determine the endpoint based on the findings at the last visit or telephone call. Four patients, however, were lost to follow-up for various reasons. The composite endpoint was major adverse cardiovascular events (MACE), including all-cause death, myocardial infarction, heart failure, recurrent angina, and target lesion revascularization, which were identified through hospitalization or phone calls.

### Sample collection

Peripheral venous blood samples were collected from each subject who was in a fasting state into a Vacutainer tube containing potassium EDTA during the baseline examination. Then, peripheral blood mononuclear cells (PBMCs) were immediately isolated from peripheral venous blood using Ficoll density gradient centrifugation. Blood samples from AMI patients were obtained immediately after coronary angiography or percutaneous coronary intervention within 48 h after admission, while blood samples from the control group were obtained after coronary angiography.

### Real-time PCR analysis of GSDMD

Total RNA was collected using TRIzol reagent according to the manufacturer’s protocol (Thermo Fisher Scientific). Two micrograms of total RNA from each sample were used to generate cDNAs using the cDNA Transcription Kit (Thermo Fisher Scientific K1622). Then, a real-time polymerase chain reaction (RT-PCR) was performed using the SYBR Premix Ex Taq Kit (TaKaRa, Japan). PCR was directly monitored using the ABI 7500 platform. All results were normalized against GAPDH (B661204; Sangon Biotech, Shanghai, China).

The primer sequences used in the study were as follows:

GSDMD: Forward primer 5′-TGGCAGGAGCTTCCACTTCT − 3′.

Reverse primer 5′-GAGGTGCTGGAGCTGTCAGA-3′.

GAPDH: Forward primer 5′-ACGGATTTGGTCGTATTG-3′.

Reverse primer 5′-TCCCGTTCTCAGCCTTG-3′.

### Statistical analysis

All values are presented as the mean ± standard deviation for variables and as the number (%) for incidence rates. Statistical analysis was performed using SPSS v.20 (SPSS Inc, Chicago, IL) and MedCalc 18.2 software (MedCalc Software, Ostend, Belgium). Statistical evaluation of the data was performed using the T test or the Wilcoxon rank sum test to compare variables between two groups, and the chi-square test was used to compare proportions. The diagnostic ability of the GSDMD level as a predictor for distinguishing AMI from non-AMI was ascertained by receiver operating characteristic (ROC) curve analysis. Spearman’s test was performed to determine the correlations between variables. Univariate and multivariate Cox regression analyses were used to assess the relationship between GSDMD level and risk of MACE after AMI. *P* value < 0.05 was considered significant.

## Results

### Clinical characteristics of the study population

The detailed demographic and clinical characteristics of 150 patients are provided in Table 1, where AMI (n = 100) contained the groups with AMI with single and multivessel vessel disease. As a result, several interrelated factors showed higher levels in the AMI group than in the control group, such as the male-to-female ratio, smoking, low-density lipoprotein-cholesterol (LDL-C), C-reactive protein (CRP), total cholesterol (TC), white blood count (WBC), left ventricular end-diastolic dimension (LEVDD) and left ventricular ejection fraction (LVEF) (*P* < 0.05). As mentioned above, most of the differential indicators were present in both single-vessel and multi-vessel lesions. However, different from single-vessel lesions, multiple lesions also showed statistical high expression in diabetes and red-cell distribution width (RDW).

**Table 1:** Baseline demographic and clinical characteristics of study participants

Characteristic	Control group (n = 50)	AMI with single vessel disease group (n = 40)	*P* value	AMI with multivessel disease group (n = 60)	*P* value
Age (years)	64.10 ± 9.88	59.90 ± 12.72	0.081	63.14 ± 12.36	0.108
Female	25 (50.0%)	10 (25.0%)	0.015**	41 (37.0%)	0.011**
Smoking	10 (20.0%)	22 (55.0%)	0.001**	40 (36.0%)	0.001**
Hypertension	30 (60.0%)	18 (45.0%)	0.160	64 (58.0%)	0.727
Hyperlipidemia	27 (54.0%)	23 (58.0%)	0.607	51 (46.0%)	0.283
Diabetes	6 (12.0%)	6 (15.0%)	0.068	24 (22.0%)	0.023*
GSDMD level	1.500 ± 0.51	2.320 ± 0.52	0.001**	2.991 ± 0.64	0.001**
LDL-C (mmol/L)	2.68 ± 1.02	3.21 ± 1.15	0.001**	2.92 ± 0.93	0.012**
HDL-C (mmol/L)	1.14 ± 0.32	1.09 ± 0.26	0.364	1.12 ± 0.27	0.347
CRP (mg/L)	3.77 ± 5.95	13.24 ± 15.43	0.001**	12.83 ± 19.35	0.001**
TC (mmol/L)	4.33 ± 1.23	5.25 ± 1.42	0.001**	4.66 ± 1.13	0.001**
TG (mmol/L)	1.60 ± 0.70	1.89 ± 2.10	0.368	1.48 ± 0.78	0.140
UA (μmol/L)	351.32 ± 86.21	363.03 ± 126.78	0.639	374.67 ± 128.95	0.083
WBC (× 10^9^/L)	6.21 ± 1.78	9.03 ± 2.69	0.001**	7.51 ± 2.52	0.001**
RDW (%)	12.95 ± 0.61	13.20 ± 1.15	0.191	13.12 ± 0.79	0.044*
Thyroxine (nmol/L)	96.40 ± 17.72	96.06 ± 17.44	0.012*	106.83 ± 24.41	0.635
LAD (mm)	38.54 ± 4.60	38.60 ± 4.72	0.952	38.80 ± 4.30	0.565
LVEDD (mm)	48.24 ± 4.74	49.53 ± 4.87	0.021*	49.75 ± 5.31	0.006**
LVEF (%)	67.24 ± 6.85	51.68 ± 9.27	0.001**	56.88 ± 11.76	0.001**

Data presented as mean ± standard deviation for variables

LDL-C, low density lipoprotein-cholesterol; HDL-C, high density lipoprotein-cholesterol; CRP, C-reaction protein; TG, triglycerides; TC, total cholesterol; UA, uric acid; WBC, white blood count; RDW, red–cell distribution width; LAD, left atrial diameter; LVEDD, left ventricular end-diastolic dimension; LVEF, left ventricular ejection fraction

*P < 0.05; ^⁎⁎^*P* < 0.01

### GSDMD was an independent biomarker of AMI

**Fig. 1:**
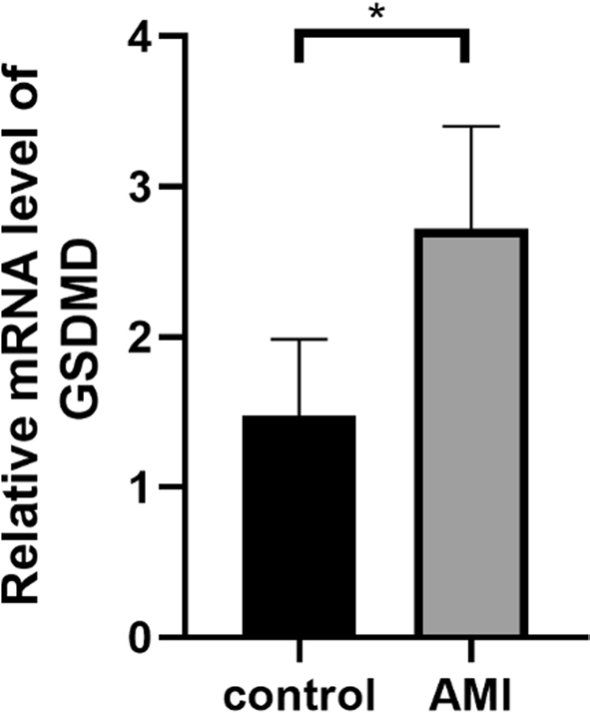
GSDMD mRNA expression level by qRT-PCR

The expression of GSDMD was measured by qRT-PCR, which showed an obvious increase in patients with AMI compared with the controls (Table 1; Fig. 1), which was consistent with previous studies [14]. As shown in Table 2, multivariate analysis revealed the correlation between GSDMD level and diabetes, LDL-C, TC, CRP, WBC, RDW and LVEF in AMI. Moreover, LDL-C and LVEF were independent factors of AMI (OR: 3.213, 95% CI 1.083–1.720, *P* = 0.047; OR: 0.795, 95% CI 0.694–0.911, *P* = 0.001; respectively). Furthermore, GSDMD may be a novel independent factor of AMI (OR: 1.365, 95% CI 1.083–1.720, *P* = 0.008).

**Table 2:** Multivariate analysis comparing risk factors associated with the GSDMD mRNA expression level in AMI

Variable	OR (95%CI)	*P* value
GSDMD level	1.365 (1.083–1.720)	0.008**
Diabetes	0.326 (0.014–7.583)	0.485
LDL-C (mmol/L)	0.495 (0.116–2.106)	0.341
TC (mmol/L)	3.213 (1.013–10.189)	0.047*
CRP (mg/L)	1.119 (0.989–1.266)	0.073
WBC (× 10^9^/L)	1.434 (0.906–2.266)	0.124
RDW (%)	1.822 (0.417–7.970)	0.426
LVEF (%)	0.795 (0.694–0.911)	0.001**

CI, confidence interval; OR, odds ratio

*P < 0.05; **P < 0.01

At the same time, considering some other variables in AMI such as diabetes, CRP and hypertension, we analyzed the correlation between GSDMD expression level and them. Data showed that GSDMD level was associated with CRP (Pearson correlation: 0.289, *P* = 0.001, Table 3) and hypertension (Pearson correlation: 0.185, *P* = 0.024), but there was no statistical correlation between GSDMD level and diabetes (Pearson correlation: −0.102, *P* = 0.215).

**Table 3:** Association of GSDMD level with diabetes, CRP and hyperlipidemia

Variable	Pearson correlation	*P* value
Diabetes	− 0.102	0.215
CRP (mg/L)	0.289	0.001**
Hyperlipidemia	0.185	0.024*

*P < 0.05; **P < 0.01

### The diagnostic ability of GSDMD in AMI

**Fig. 2:**
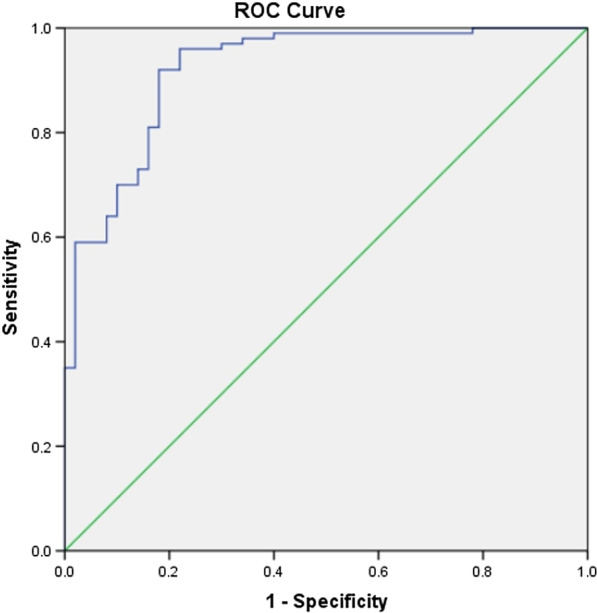
Receiver operating characteristic (ROC) curve analysis of the GSDMD expression level in AMI

**Fig. 3:**
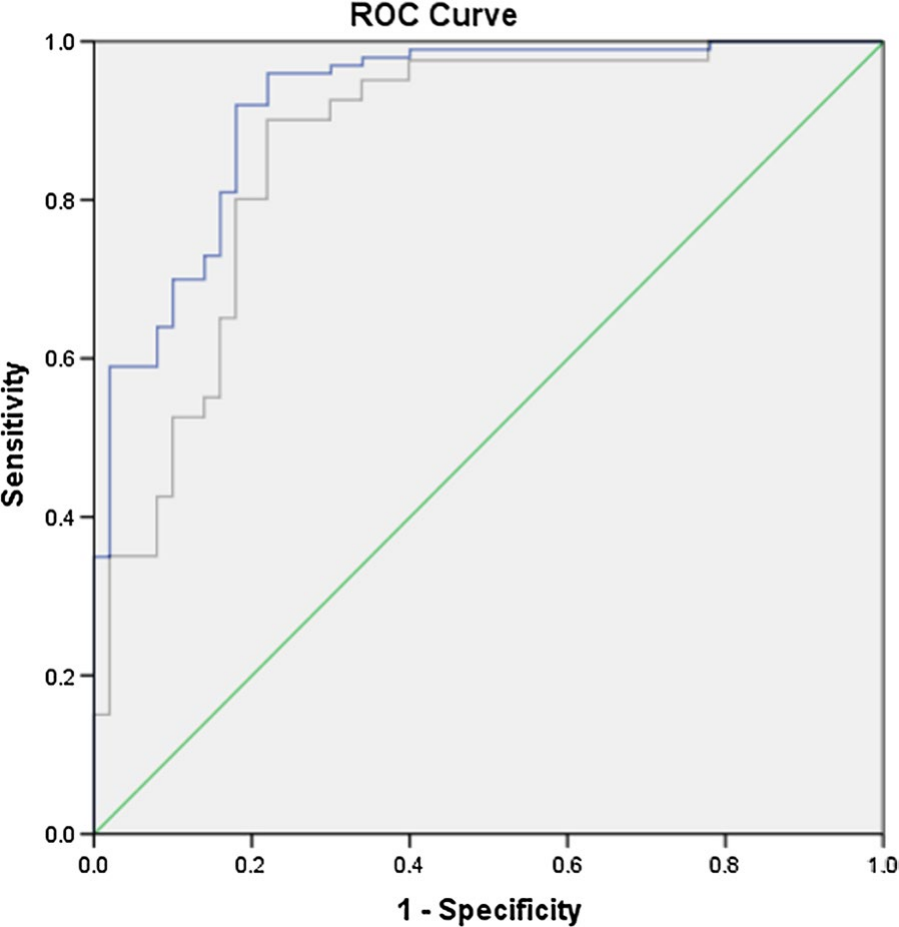
Receiver operating characteristic (ROC) curve analysis of the GSDMD expression level in AMI with single vessel and multivessel disease

ROC curve analysis was used to describe the diagnostic ability of GSDMD in AMI, especially in single or multivessel disease (Table 4). The GSDMD level had considerable predictability for AMI (AUC = 0.921, 95% CI 0.875–0.968) (Fig. 2). Moreover, the GSDMD level also showed a high level of predictability in the group with AMI with single vessel disease (AUC = 0.866, 95% CI 0.791–0.941) and the group with AMI with multivessel disease (AUC = 0.958, 95% CI 0.926–0.991) (Fig. 3). The GSDMD level demonstrated better psychometric screening properties for a cut-off score of 2.032 in the group with AMI with multivessel disease, with a sensitivity of 100.0% and a specificity of 82.0%. Moreover, Table 5 showed the comparison of the two ROC curves in the groups with AMI with single and multivessel disease, where the difference between areas was 0.092 (*P* = 0.0271). These results confirmed that the GSDMD level may be effective in the clinical diagnosis of AMI.

**Table 4:** Results of ROC curve analysis comparing the GSDMD level for the accuracy in predicting the number of vessel diseases in AMI

Parameter	AUC	95% CI	Cutoff	Sensitivity (%)	Specificity (%)	Youden index
AMI	0.921	0.875–0.968	1.793	0.960	0.780	0.740
AMI with single vessel disease	0.866	0.791–0.941	1.792	0.900	0.780	0.680
AMI with multivessel disease	0.958	0.926–0.991	2.032	1.000	0.820	0.820

**Table 5:** Comparison of the two ROC curves with AMI with vessel disease

Scales	Difference between areas	SE	Z statistic	*P* value
AMI with single vessel disease–multivessel disease	0.092	0.0416	2.210	0.0271

SE, standard error

### GSDMD related to the MACE after AMI

Patients enrolled in this study were followed up for 360 days, with four patients lost to follow-up and twenty with recurring MACE. The univariate and multivariate Cox regression analyses revealed that the GSDMD level and diabetes significantly increased the risk of MACE after AMI (GSDMD: hazard ratio [HR] 1.193, 95% CI 1.126–1.264 and HR 1.263, 95% CI 1.120–1.425; diabetes: HR 12.000, 95% CI 4.012–35.894 and HR 4.986, 95% CI 1.015–24.496; Table 6). Interestingly, LDL-C showed a risk increase with MACE in the univariate Cox regression analysis (HR 1.587, 95% CI 1.107–2.275) but not in the multivariate analysis.

**Table 6:** The correlations of the GSDMD mRNA expression level with major adverse cardiovascular events after acute myocardial infarction using the uni‐ and multivariate Cox analysis

Factor	Univariate Cox		Multivariate Cox	
	HR (95%CI)	*P*	HR (95%CI)	*P*
Age	1.011 (0.979–1.044)	0.509	0.979 (0.944–1.016)	0.261
GSDMD	1.193 (1.126–1.264)	0.001**	1.263 (1.120–1.425)	0.001**
LDL-C	1.587 (1.107–2.275)	0.012*	0.981 (0.608–1.585)	0.939
Hypertension	1.500 (0.613–3.670)	0.374	4.954 (0.925–26.536)	0.062
Diabetes	12.000 (4.012–35.894)	0.001**	4.986 (1.015–24.496)	0.048*
Hyperlipidemia	2.433 (0.935–6.330)	0.068	0.271 (0.044–1.0682)	0.161

*P < 0.05; **P < 0.01

## Discussion

In total, this study indicated that the GSDMD expression level was closely related to AMI. With a significant increase in PBMCs in patients with AMI, GSDMD was further found to be an independent biomarker of AMI by multivariate analysis. Moreover, GSDMD showed promise in diagnosing AMI (AUC = 0.921, 95% CI 0.875–0.968), especially in multivessel disease (AUC = 0.958, 95% CI 0.926–0.991) with a sensitivity of 100.0% and a specificity of 82.0%. Furthermore, univariate and multivariate Cox regression analyses suggested that the GSDMD level and diabetes were significantly related to the risk of MACE after AMI.

Widely expressed in different types of cells and tissues, GSDMD is part of the Gasdermin superfamily in humans, a novel group of genes that consist of GSDMA, GSDMB, GSDMC and GSDMD, as well as the Gasdermin-related genes (GSDME and pejvakin) [15]. GSDMD consists of an N-terminal pore-forming domain and a C-terminal inhibitory domain, and the N-terminus is the main functional part involved in pyroptosis [16, 17]. Evidence that pyroptosis mediated by GSDMD is involved in many inflammation-related diseases has been gradually excavated [18, 19]. And in the cardiovascular field, GSDMD is also beginning to show its unique value. In acute coronary syndrome (ACS), the overexpression or inhibition of IRF-1 effectively modulated caspase-1 activation, macrophage lysis and GSDMD expression, suggesting that IRF-1 potently activates ox-LDL-induced macrophage pyroptosis [20]. Moreover, studies have demonstrated that the NF-κB-GSDMD axis functions as a bridge between oxidative stress and NLRP3 inflammasome-mediated cardiomyocyte pyroptosis [21]. Suppression of oxidative stress alleviated pyroptosis in H9c2 cells and reduced NF-κB and GSDMD activity, characterized by LDH release and NLRP3 inflammasome activation in H9c2 cells under oxygen-glucose deprivation conditions [22]. These studies suggested that GSDMD indeed participates in the development of AMI. Consistent with these results, our study further showed that the expression of GSDMD was significantly increased in Chinese patients with AMI. At the same time, the AMI patients we focused on were all suffered from occlusion of atherosclerotic plaque and eligible for Percutaneous Coronary Intervention (PCI).

The pyroptosis of AMI has received much attention in recent years. Surprisingly, we revealed that GSDMD level was positively correlated with MACE risk after AMI, suggesting the potential of GSDMD as a prognostic assessment in addition to its diagnostic value for AMI. In addition, diabetes has the same positive association with MACE. Diabetes is a common concomitant disease in patients with AMI, and its burden on patients is keeping increasing [23]. But it remains controversial the harmful effect of diabetes on thetality in AMI patients. A multi-center study indicated that ST-segment elevation myocardial infarction (STEMI) patients with diabetes showed a 22% higher 1-year mortality than those without diabetes in a cohort of 62,036 patients from 55 countries [24]. However, some studies have observed no significant correlation between diabetes and AMI mortality.

Nevertheless, several limitations should be acknowledged. First and foremost, the number of Chinese patients with AMI enrolled in this study is limited, so a large cohort is required in future studies. As a cross-sectional study, this study cannot demonstrate the causality between GSDMD expression level and AMI, so further studies are required to prove it.

## Conclusion

This study indicated that the GSDMD expression level in PBMCs was elevated in AMI patients and was closely associated with the pyroptosisof AMI.

## Data Availability

All data generated or analysed during this study are included in this published article.

## Abbreviations

AMI: Acute myocardial infarction
GSDMD: Gasdermin D
ESCC: Oesophageal squamous cell carcinoma
ECGs: Electrocardiograms
MACE: Major adverse cardiovascular events
PBMCs: Peripheral blood mononuclear cells
ROC: Receiver operating characteristic
LDL-C: Low density lipoprotein-cholesterol
CRP: C-reactive protein
TC: Total cholesterol
WBC: White blood count
LEVDD: Left ventricular end-diastolic dimension
HDL-C: High density lipoprotein-cholesterol
TG: Triglycerides
UA: Uric acid
RDW: Red-cell distribution width
LAD: Left atrial diameter
